# Multi-Omics Profiling Unveils the Complexity and Dynamics of Immune Infiltrates in Intrahepatic Cholangiocarcinoma

**DOI:** 10.3390/biology13100816

**Published:** 2024-10-11

**Authors:** Xuan Li, Yan Wang, Renchu Guan, Nan Sheng, Shuangquan Zhang

**Affiliations:** 1 Key Laboratory of Symbolic Computation and Knowledge Engineering of Ministry of Education, College of Computer Science and Technology, Jilin University, Changchun 130012, China; lxuan18@mails.jlu.edu.cn (X.L.); guanrenchu@jlu.edu.cn (R.G.); shengnan21@mails.jlu.edu.cn (N.S.); 2 School of Cyber Science and Engineering, Nanjing University of Science and Technology, Nanjing 210094, China

**Keywords:** intrahepatic cholangiocarcinoma, machine learning, multi-omics data, immune cells

## Abstract

**Simple Summary:**

ICC is a highly heterogeneous malignancy. Within the TME, there is limited knowledge of immune cells that are closely associated with and interact with ICC. This study used machine learning methods to conduct a more comprehensive analysis of multi-omics data and found that the effect of APCs on activating NK cells was weakened, while the effect of activating T cells was enhanced. At the same time, four distinct subpopulations were identified, thereby revising and constructing an immune sensing and response model for ICC.

**Abstract:**

Intrahepatic cholangiocarcinoma (ICC) is a highly heterogeneous malignancy. The reasons behind the global rise in the incidence of ICC remain unclear, and there exists limited knowledge regarding the immune cells within the tumor microenvironment (TME). In this study, a more comprehensive analysis of multi-omics data was performed using machine learning methods. The study found that the immunoactivity of B cells, macrophages, and T cells in the infiltrating immune cells of ICC exhibits a significantly higher level of immunoactivity in comparison to other immune cells. During the immune sensing and response, the effect of antigen-presenting cells (APCs) such as B cells and macrophages on activating NK cells was weakened, while the effect of activating T cells became stronger. Simultaneously, four distinct subpopulations, namely $B^{Lp}$, ${Macrophages}^{Lp}$, $B^{Hn}$, and $T^{Hn}$, have been identified from the infiltrating immune cells, and their corresponding immune-related marker genes have been identified. The immune sensing and response model of ICC has been revised and constructed based on our current comprehension. This study not only helps to deepen the understanding the heterogeneity of infiltrating immune cells in ICC, but also may provide valuable insights into the diagnosis, evaluation, treatment, and prognosis of ICC.

## 1. Introduction

In addition to being the second most common primary liver cancer, ICC is a highly lethal hepatobiliary adenocarcinoma [1]. Its incidence continues to increase worldwide and its prognosis is extremely poor. This tumor exhibits a significant desmoplastic nature and is encompassed by an immune microenvironment characterized by a dense aggregation of inflammatory cells and matrix components [1,2,3,4]. Tumor tissue consists not only of tumor cells, but also of a heterogeneous microenvironment with immune cells and stromal cells [5]. Throughout the various stages of cancer progression, ranging from the initiation of tumor formation to the spread of cancer cells to distant sites, the intricate interplay between tumor cells and their surrounding microenvironment can exert either a suppressive or supportive effect on tumor growth [6,7]. It is widely acknowledged in the scientific community that immunosurveillance is a process by which immune cells residing within the TME detect and eliminate malignant cells through diverse immunological mechanisms [8].

Within the TME, there are a variety of innate immune cells, such as macrophages and NK cells. They significantly influence the occurrence of ICC. Tumor-associated macrophages (TAMs) represent a significant infiltrating immune cell population within the TME [4,9]. Elevated macrophage density in the tissue has been linked to unfavorable prognostic outcomes in individuals diagnosed with ICC [4,10]. Macrophages that are phenotypically polarized towards M2 exhibit tumor-promoting behavior, which is associated with adverse prognosis and the occurrence of metastasis in ICC [11]. Macrophages actively contribute to tumor growth by engaging in reciprocal communication with ICC cells and releasing an array of inflammatory, growth, and proliferation factors to facilitate tumor progression [12,13].

NK cells possess the capacity to identify and eliminate malignant cells by means of the release of cytotoxic granules [14]. Through the modulation of an extensive array of activating and inhibitory immune receptors expressed on their surface, NK cells are capable of discerning and reacting to neoplastic cells in nearby areas. In the liver, NK cells constitute approximately 30~40% of all lymphocytes [15]. Nevertheless, there exists a dearth of knowledge regarding the characteristics and behavior of NK cells in ICC.

The adaptive immune cells within the TME are mainly B cells and T cells. In ICC, CD4+ tumor-infiltrating lymphocytes (CD4+ TILs) predominantly localize in the peritumoral region, while CD8+ tumor-infiltrating lymphocytes (CD8+ TILs) are primarily found within the intratumoral tissues [16,17]. Numerous investigations have consistently demonstrated that increased levels of CD4+ TILs and CD8+ TILs in ICC are correlated with improved overall patient survival, reduced risk of lymph node metastasis, and reduced extent of venous and perineural tissue invasion [18,19,20,21]. Conversely, a scarcity of CD8+ TILs is associated with inferior overall survival outcomes [22].

B cells can regulate immune responses through a variety of mechanisms. These include the inhibition of T cells differentiation into Th1 and Th17 cells, as well as diminishing the synthesis of proinflammatory cytokines by CD4+ effector T cells. Additionally, B cells may suppress cytotoxic CD8+ T cell responses [23]. On the positive side, B cells have been found to negatively regulate tumor growth in multiple cancer types. In some cancer cases, CD20+ B cell TILs detected in tumor tissue are associated with improved survival and reduced recurrence rates. The mechanisms that contribute to B cell-mediated antitumor immunity may encompass the secretion of effector cytokines by B cells. These cytokines have the potential to influence the polarization of T cells towards either Th1 or Th2 responses, as well as facilitate T cell responses through their function as APCs [23]. The efficacy of immunotherapy heavily relies on a comprehensive comprehension of immune cells and immune-related markers within the TME [24]. While immunotherapy has demonstrated success in various cancer types, such as ICC, our understanding of the immune status and molecular attributes of the TME remains constrained [25,26,27,28].

By utilizing large-scale genomics and transcriptomics, researchers can effectively identify crucial gene mutations and aberrant signaling pathways in ICC [29,30,31,32,33]. It is important to note that these investigations frequently depend on data obtained from batch analyses, which impose restrictions on their capacity to precisely capture tumor heterogeneity. Nevertheless, the application of single-cell RNA sequencing (scRNA-seq) technology has provided great help in observing the diversity of cells and their intercellular communication by employing single-cell resolution [34,35,36]. Consequently, scRNA-seq has become a novel approach for studying the intricate tumor and immune landscapes in various cancer types [35,37,38]. By using this technique, we can gain a greater understanding of cellular heterogeneity and identify promising molecular targets that can be used for antitumor therapies [39]. Despite the existence of numerous scRNA-seq studies that have highlighted the intricacy of the TME in ICC, the absence of integrated transcriptomics samples and corresponding clinical phenotypes for classification may compromise the accurate representation of tumor diversity [40].

This study aimed to investigate the diversity and characteristics of infiltrating immune cells within the TME of ICC. We performed a more comprehensive analysis of multi-omics data. From the perspective of transcriptomic analysis, single-cell analysis, and joint analysis, we used statistical and machine learning methods to identify two different clusters of immunoactivity. The B cells, macrophages, and T cells in the infiltrating immune cells of ICC were relatively active. We identified four subpopulations of ICC-infiltrating immune cells, from which we constructed immune-related prognostic labels corresponding to them. Finally, drawing upon our existing comprehension of the immune sensing and response exhibited by ICC, we proceeded to revise and construct it, ultimately presenting a novel ICC immune sensing and response model devised by our team. The utilization of multi-angle analyses has yielded valuable insights into the immune status of ICC and the prospective clinical implementation of immunotherapy.

## 2. Materials and Methods

### 2.1. Materials

We obtained a total of 44 cases of ICC transcriptomics samples and their corresponding clinical phenotypes from the GDC Data Portal and the UCSC Xena Portal. This dataset comprised 35 primary cancer samples and 9 paracancer samples. From the NCBI Gene Expression Omnibus (GEO) database, we collected data on 55,359 cells derived from 4 treatment-naive ICC samples, 1 recurrent ICC sample, 3 adjacent tissue samples, and 1 normal tissue sample. We obtained the LM22 matrix gene set, comprising 22 distinct immune cell types and 547 genes associated with immune function, from the CIBERSORT website. This dataset was used to estimate the abundance of immune cells within the sample. We analyzed immune infiltration in the tumor microenvironment by collecting 16 immune-related gene sets containing 929 genes from TISCH2.

### 2.2. Methods

**Figure 1:**
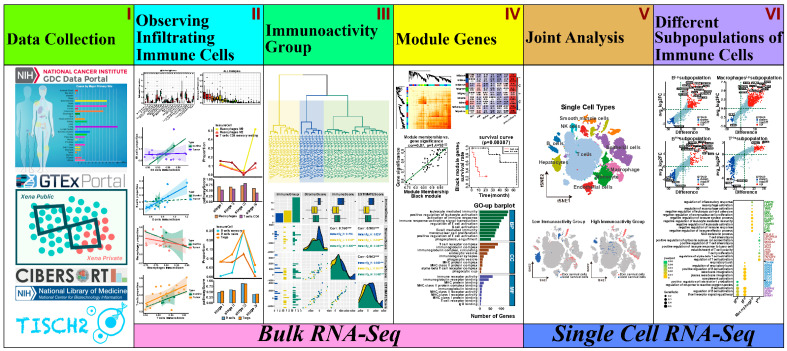
Process framework diagram. (**I**) We downloaded and collected data from GDC, UCSU Xena Portal, NCBI, CIBERSORT, and TISCH2. (**II**) Differential analysis, immune cell abundance analysis, correlation analysis, and GSEA analysis were performed on the infiltrating immune cells of the samples. The activity of B cells, macrophages, T cells, and NK cells was analyzed. (**III**) We classified the samples based on their immunoactivity and conducted a comparative analysis between the group with low immunoactivity and the group with high immunoactivity. (**IV**) Key genes were screened out through WGCNA processing among differential genes. (**V**) Joint analysis of bulk data and single-cell data to find out immune cell subpopulations. (**VI**) Analysis and comparison of individual subpopulations of immune cells.

As shown in Figure 1, the framework of our analysis process consists of “Data Collection”, “Observe Infiltrating Immune Cells”, “Immunoactivity Group”, “Module Genes”, “Joint Analysis”, and “Different Subpopulations of Immune Cells” modules.

#### 2.2.1. The Quantification of Infiltrating Immune Cells

We estimated the proportion of infiltrating immune cell in each ICC sample using the Cibersort analysis tool (R script version 1.03), which incorporates the LM22 gene set consisting of 22 immune cell types and 547 immune-related genes, available on the CIBERSORT website. We used the Spearman correlation coefficient to assess the correlation between gene expression and the level of immune cell infiltration [41]. It should be noted that the proportion of infiltrating immune cells assessed by Cibersort is a relative proportion [42].

#### 2.2.2. ssGSEA Signature Score

In order to calculate ssGSEA scores, we used the Gene Set Variation Analysis (GSVA) R package (version 1.42.0). The ssGSEA algorithm assigns enrichment scores to each sample that characterize the joint analysis of a set of genes. The ssGSEA signature score was utilized to quantify the level of immunoactivity in the samples. By integrating this score with the sum of divergences of squares method, the samples were categorized into groups representing “low-moderate-high immunoactivity”. A higher ssGSEA score indicates a more immunocompetent sample [43,44].

#### 2.2.3. Estimate

Using the Estimate R package (version 1.0.13), we were able to analyze the tumor microenvironment of samples in the low-moderate-high immunoactivity groups, yielding StromalScore, ImmuneScore, and ESTIMATEScore, respectively [45].

#### 2.2.4. Comparison of Immunoactivity

We designed a method to estimate and compare immunoactivity by looking at the magnitude of the change from one state to another (see Supplementary Materials).

#### 2.2.5. Statistical Analysis of Differences

A differentiating analysis was performed for the 9 samples from the low immunoactivity group versus the 7 samples from the high immunoactivity group using the DESeq2 R package (version 1.34.0).

#### 2.2.6. Principal Component Analysis

We removed the meaningless gene expression, leaving 46,762 genes. Subsequently, principal component analysis (PCA, see Supplementary Materials) and clustering were carried out. We found that the weight interference of non-differential genes would dilute and weaken the main weight of differential genes in the clustering process, while PCA could ensure and evaluate the main role of differential genes in the clustering process [46].

#### 2.2.7. Multiple Correspondence Analysis

Multiple correspondence analysis (MCA) enabled us to cluster data that had been processed. PC1, PC2, and PC3 were selected as the three dimensions of spatial clustering. This analytical approach was employed to ascertain the reliability of the selected principal components by assessing whether the cumulative contribution of the variances of PC1, PC2, and PC3 attained or surpassed 75% [47,48].

#### 2.2.8. Weighted Correlation Network Analysis

We performed a cluster analysis of gene co-expression using the WGCNA R package (version 1.72-1). Specifically, the algorithm utilized a total of 16 samples from the low-high immunoactivity group and 1815 genes. Initially, we constructed a directional Pearson correlation matrix to form a signed co-expression network. This matrix was then converted into an adjacency matrix for subsequent cluster analysis [49,50]. To define and merge co-expression clusters, the hybrid dynamic tree-cutting method was employed [51]. By evaluating the first principal component, referred to as the eigengene, for each co-expression cluster, a consensus trend was determined. To determine the extent of cluster affiliation, the Pearson correlation coefficient was computed between each gene and its corresponding consensus within each module. Genes exhibiting a correlation coefficient of at least 0.75 were allocated to the co-expression clusters displaying the highest degree of correlation.

#### 2.2.9. Cox Proportional Hazards Model

Based on a Cox proportional hazards model (Cox regression), we performed multivariate survival analyses, and we considered the results to be statistically significant when *p* value < 0.05.

#### 2.2.10. survivalROC

The R package survivalROC (version 1.0.3.1) provides the function “roc” for performing ROC analyses [52]. The gene modules selected by WGCNA were placed in the low immunoactivity group and also in the high immunoactivity group, and then the survival status of the two groups was compared using survivalROC.

#### 2.2.11. Gene Ontology

A pathway enrichment analysis and subsequent biological interpretation were conducted utilizing Gene Ontology (GO) terms categorized into three distinct domains: biological process, cellular component, and molecular function [53].

#### 2.2.12. Kyoto Encyclopedia of Genes and Genomes

The Kyoto Encyclopedia of Genes and Genomes (KEGG) database (https://www.genome.jp/kegg/, accessed on 27 December 2023) was utilized as a reference, we identified significantly enriched signaling pathways and set *p* value < 0.05 and q value < 0.05 to screen these pathways.

#### 2.2.13. Seurat

For cell clustering, we used the Seurat (https://satijalab.org/seurat/ accessed on 1 May 2024) tool. When creating the Seurat object, we filtered cells based on the condition that each gene must be detected in at least 100 cells and each cell must express at least 300 gene features. Then, further processing, cells with less than 500 gene features and more than 5% of the mapped reads to the mitochondrial genome were also filtered out. We used the “LogNormalize” method to adjust all gene expression values of each cell according to the default scale factor of 10,000, and then logarithmically transformed the scaled expression values [54]. After filtering and normalization, a total of 36,558 cells were chosen to improve the quality of subsequent single-cell analysis. The gene expression values of cells were normalized after processing with the “ScaleData()” function. To create a graphical representation of t-distributed stochastic neighbor embedding (t-SNE), we used the Seurat package (version 4.3.0) to perform the following operations: identification of the top 2000 genes with the greatest variability in the dataset, PCA, JackStraw (https://cran.r-project.org/web/packages/jackstraw/ accessed on 1 May 2024) analysis, and SNN-Cliqinspired clustering [55]. In terms of identifying and annotating cell clusters, we used the “FindClusters()” function to identify based on marker genes, and then used the Single-cell Recognition (SingleR, https://comphealth.ucsf.edu/ accessed on 20 May 2024) package to annotate the identified cell clusters with cell types. Finally, we used the ClusterProfiler package to perform GO analysis of genes.

#### 2.2.14. Scissor

The Scissor tool (https://github.com/sunduanchen/Scissor, accessed on 1 July 2024) integrates single-cell data, transcriptomics samples data, and clinical survival data in a comprehensive analysis to identify unique cell subpopulations [56]. It employs the Pearson correlation to assess the similarity between single-cell and transcriptomics samples data, incorporating clinical phenotypes to construct a correlation matrix. Subsequently, Scissor employs a Cox regression model within the correlation matrix, applying sparsity penalty and graph regularization techniques to identify single cells with high confidence. An identified cell can be categorized as a poor survival cell or a good survival cell, thereby indicating its positive or negative correlation with the phenotype under investigation [57].

## 3. Results

### 3.1. APCs in ICC Have a Stronger Effect on T Cell Activation

**Figure 2:**
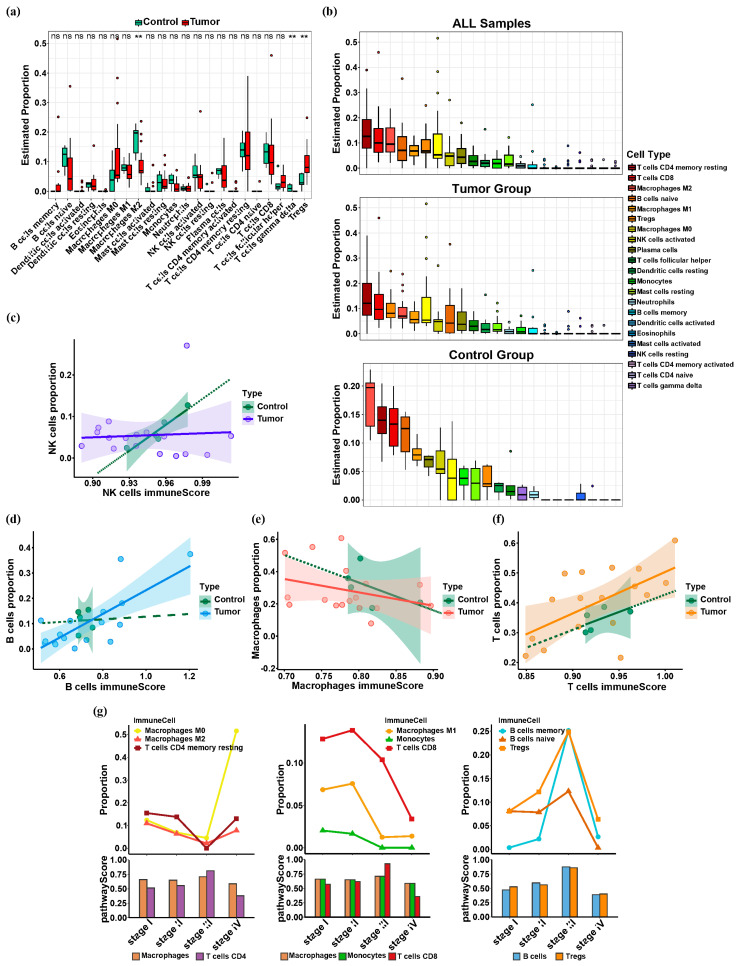
The status of APCs and T cells in ICC. (**a**) There are significant differences in macrophages M2, Tregs, and T cells gamma delta between the tumor group and the control group. Where *p* value > 0.05, ns; *p* value ≤ 0.01, **. (**b**) The ranking of immune cell proportions across all samples, tumor group, and control group. The combined sorting results from these three images collectively suggest that the proportions of B cells, macrophages, and T cells are relatively elevated, whereas the proportion of NK cells is not notably prominent. (**c**) Changes in the immunoactivity of NK cells. $R_{Tumor}^{2}$ = 0.43, $p\;{value}_{Tumor}$ = 0.002; $R_{Control}^{2}$ = 0.74, $p\;{value}_{Control}$ = 0.017; ∆k = −0.013. (**d**) Changes in the immunoactivity of B cells. $R_{Tumor}^{2}$ = 0.62, $p\;{value}_{Tumor}$ = 6.8 × 10^−5^; $R_{Control}^{2}$ = 0.87, $p\;{value}_{Control}$ = 0.004; ∆k = 0.002. (**e**) Changes in the immunoactivity of macrophages. $R_{Tumor}^{2}$ = 0.76, $p\;{value}_{Tumor}$ = 1.6 × 10^−6^; $R_{Control}^{2}$ = 0.84, $p\;{value}_{Control}$ = 0.007; ∆k = 0.887. (**f**) Changes in the immunoactivity of T cells. $R_{Tumor}^{2}$ = 0.94, $p\;{value}_{Tumor}$ = 3.1 × 10^−11^; $R_{Control}^{2}$ = 0.99, $p\;{value}_{Control}$ = 2.2 × 10^−5^; ∆k = 0.065. (**g**) In the context of tumor progression, a positive correlation exists between T cells and APCs across the four stages. The line chart’s y-axis represents the proportion of immune cells, while the histogram’s y-axis measures pathwayScore, a measure of immune cell activity.

The proportions of immune cells were analyzed in 44 ICC samples (35 primary cancer samples and nine paracancer samples) from the GDC dataset using the Cibersort analysis tool. The calculation results yielded samples with *p* value < 0.05, which were subsequently categorized into two groups: the Control Group consisting of five paracancer samples, and the Tumor Group comprising 17 primary cancer samples. Based on Wilcox test calculations, significant differences in macrophages M2, Tregs, and T cells gamma delta were observed between the two groups (Figure 2a).

We ranked the abundance of 22 immune cells in all samples, primary cancer samples, and paracancer samples, respectively. Immune cells with a significant proportion of abundance were screened. The median proportions of T cells CD4 memory resting, T cells CD8, macrophages M2, and B cells naive were high in all samples. The median proportions of T cells CD4 memory resting, T cells CD8, Tregs, and macrophages M2 were higher in the primary cancer samples. The median proportions of macrophages M1, macrophages M2, T cells CD4 memory resting, T cells CD8, and B cells naive were higher in the paracancer samples (Figure 2b). The absence of discernible proportions of NK cells was observed across all cases. Our analysis of immune cell abundance in ICC revealed a generally high proportion of macrophages, B cells, and T cells, while the proportion of NK cells remained inconspicuous.

Across each stage of the tumor, T cells and APCs were positively correlated in terms of abundance changes and pathway strength changes (Figure 2g). It can be observed that when the immunoactivity of NK cells in a healthy state is transformed into the immunoactivity in a cancerous state, the slope of the regression line decreases, indicating that the immunoactivity of NK cells decreases (Figure 2c). When the immunoactivity of B cells, macrophages, and T cells in a healthy state is transformed into the immunoactivity in a cancerous state, the slope of the regression line increases, indicating that their immunoactivity is enhanced (Figure 2d–f). Therefore, we suggest that in the stage where APCs act on NK cells and T cells, the effect of APCs on activating NK cells is weakened, whereas the effect on activating T cells is strengthened.

### 3.2. Infiltrating Immune Cells from ICC Show Varying Immunoreactivity Levels

**Figure 3:**
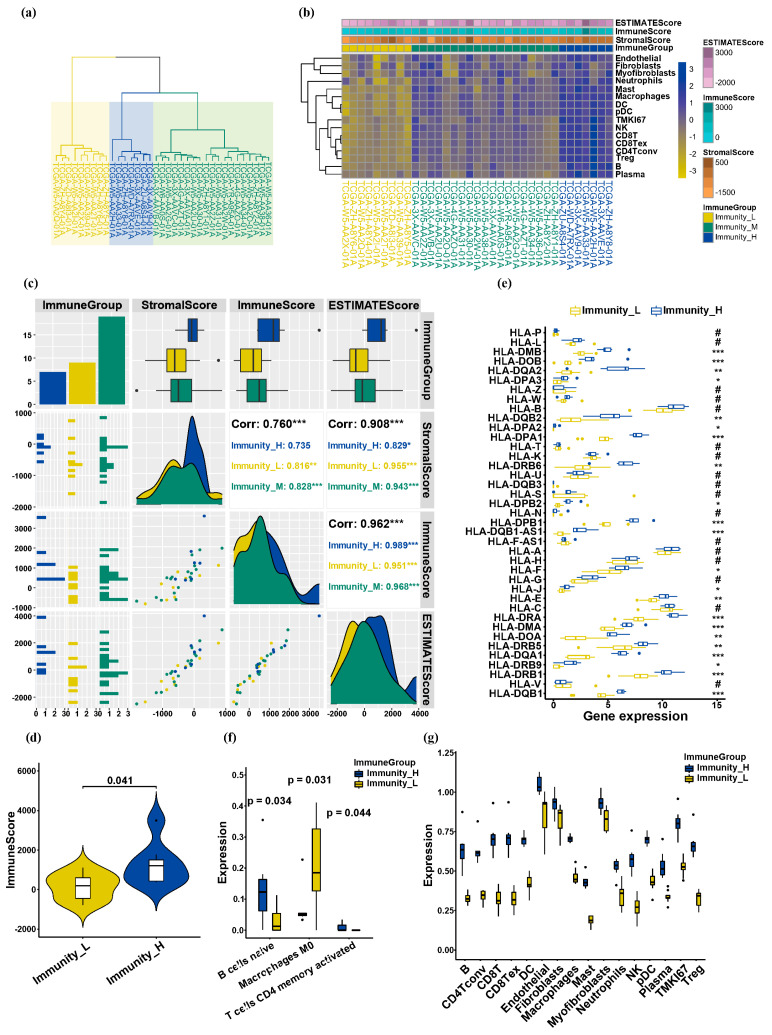
Grouping of ICC samples. (**a**) The primary cancer samples were categorized into three groups based on the level of immune infiltration within the TME: the low immunoactivity group, the moderate immunoactivity group, and the high immunoactivity group. (**b**) Estimate tool calculates StromalScore, ImmuneScore, and ESTIMATEScore of each immunoactivity group. (**c**) The correlations between each group of samples and StromalScore, ImmuneScore, and ESTIMATEScore. (**d**) There exists a notable disparity between the groups exhibiting low and high immunoactivity. (**e**) The comparison of high-low immunoactivity groups in terms of HLA-related gene expression. Where *p* value > 0.05, #; *p* value ≤ 0.05, *; *p* value ≤ 0.01, **; *p* value ≤ 0.001, ***. (**f**) On the LM22 immune-related gene set, there are differences between the low immunoactivity group and the high immunoactivity group regarding B cells naive, macrophages M0, and T cells CD4 memory activated. (**g**) On the TISCH2 immune-related gene set, the above two groups have significant differences in immune cells such as B cells, T cells, and macrophages. B: *p* value = 4.5 × 10^−4^; CD4Tconv: *p* value = 3.3 × 10^−5^; CD8T: *p* value = 1.1×10^−5^; CD8Tex: *p* value = 1.2 × 10^−5^; DC: *p* value = 2.2 × 10^−8^; Endothelial: *p* value = 0.00169; Fibroblasts: *p* value = 0.02595; Macrophages: *p* value = 1.8 × 10^−8^; Mast: *p* value = 1.2 × 10^−7^; Myofibroblasts: *p* value = 0.00489; Neutrophils: *p* value = 1.5 × 10^−4^; NK: *p* value = 1.4 × 10^−5^; pDC: *p* value = 4.9 × 10^−8^; Plasma: *p* value = 6.4 × 10^−4^; TMKI67: *p* value = 2.4 × 10^−5^; Treg: *p* value = 5.4 × 10^−6^.

Based on the immunoactivity obtained previously using ssGSEA, the immunoactivity expression values of the 35 primary cancers were calculated as the sum of squared differences and clustered into “low immunoactivity group” (nine samples), “moderate immunoactivity group” (19 samples), and “high immunoactivity group” (seven samples) (Figure 3a). Using the Estimate tool to calculate StromalScore, ImmuneScore, and ESTIMATEScore for each immunoactivity group, we found that the immunoactivity was positively correlated with these scores, and the corresponding tumor purity was negatively correlated (Figure 3b). Then, we calculated the correlation between the samples of each group and StromalScore, ImmuneScore, and ESTIMATEScore. At the same time, the correlation between StromalScore, ImmuneScore, and ESTIMATEScore was calculated, and the correlation was found to be significant (Figure 3c). We extracted samples from the “low immunoactivity group” and “high immunoactivity group”, and found a significant difference between the “low immunoactivity group” and “high immunoactivity group” with *p* value < 0.05 for the comparison of ImmuneScore (Figure 3d). In terms of human leukocyte antigen (HLA)-related gene expression, the comparison of high-low immunoactivity groups showed that 22 of the 39 HLA-related genes had significant differences (Figure 3e). Comparing the above two groups on the LM22 immune-related gene set, there are differences between the two groups regarding B cells naive, macrophages M0, and T cells CD4 memory activated (Figure 3f). In addition, the analysis within the TISCH2 immune-related gene set also revealed obvious differences in immune cells such as B cells, T cells, and macrophages in the two groups of samples (Figure 3g). Consequently, an examination of the TME in primary ICC cancer samples reveals a negative correlation between the level of immune cell infiltration and tumor purity, aligning with prior knowledge. Furthermore, our comprehensive investigation demonstrates that infiltrating immune cells in ICC possess distinct immunoreactivity, including B cells, T cells, macrophages, and others.

### 3.3. Key Genes from Modularized Immunoactivity Groups

**Figure 4:**
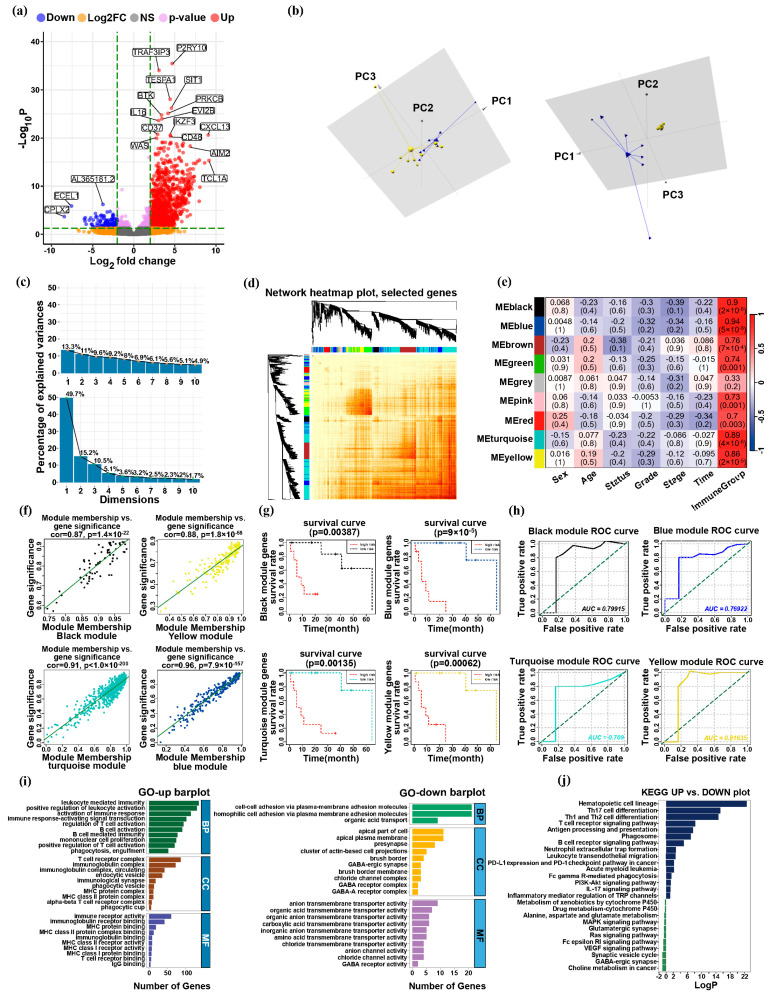
Modularization of immune activity groups. (**a**) Differential analysis of low-high immunoactivity groups. (**b**) The three principal components PC1, PC2, and PC3 were constructed into spatial coordinates. The left picture in the figure is the clustering effect before filtering, and the right picture is the clustering effect after filtering. (**c**) The figure displays the cumulative contribution of the variance of the first three principal components before and after screening, as determined through MCA analysis. The top section represents the cumulative contribution prior to screening, while the bottom section represents the cumulative contribution after screening surpasses the threshold of 75%. (**d**) WGCNA analysis was performed on 1815 significantly differentially expressed genes. Different colors represent different gene co-expression modules, and above each module is the corresponding gene clustering dendrogram. (**e**) Genes in modules are associated with immunoactivity groupings. (**f**) The black, blue, turquoise, and yellow modules showed features that were highly correlated with immunoactivity grouping. (**g**) Multivariate regression analysis of 16 samples was performed using a Cox proportional hazard model to explore the risk associations between different immunoactivity groups. (**h**) ROC curves validated the use of the Cox regression model in the immunoactivity groups. (**i**) GO analysis. (**j**) KEGG analysis.

A differential analysis was conducted comparing the “low immunoactivity group” with the “high immunoactivity group”, and screened out 1623 up-regulated genes and 192 down-regulated genes (Figure 4a). WGCNA analysis was performed on 1815 significantly differentially expressed genes. To evaluate and quantify similarity between gene expression, we used the Pearson correlation coefficient and constructed a similarity matrix based on it. Subsequently, the topological overlap count of this matrix was utilized to construct the gene co-expression network. The dynamic shear tree method was used for hierarchical clustering, and several clusters were obtained (Figure 4d). The genes in the module were then correlated with the phenotype and immunoactivity grouping of the samples (Figure 4e). Correlation between the two is more than 85%, which makes us believe there is a strong relationship between them. It can be observed that the Black Module (70 genes), Blue Module (282 genes), Turquoise Module (781 genes), and Yellow Module (177 genes) are very strongly correlated with immunoactivity grouping. Through regression analysis, it was found that the genes in the module were significantly correlated with the corresponding module (Figure 4f). Multivariate regression analysis of 16 samples was performed using Cox proportional hazard model to assess the risk differences between different immunoactivity groups. As for the genes in the above four modules, we performed survival analysis on the samples (Figure 4g), and the results showed that the *p* value < 0.05, indicating that the genes in the four modules had a high correlation with the survival of the samples. The prediction of five-year survival time using the survivalROC curve in the low-high immunoactivity group using the Cox regression model on the genes of the four modules showed good results (Figure 4h). Thus, the genes within the four modules were subjected to screening, revealing a co-expression relationship among the genes within each module, resulting in a total of 1310 genes.

Sixteen samples in the low-high immunoactivity groups were randomly shuffled, and we performed PCA by 1310 genes on the samples. The three principal components PC1, PC2, and PC3 were constructed into spatial coordinates, and the 16 samples were clustered, and the clustering effect was obvious (Figure 4b). By conducting MCA analysis, it was determined that the combined contribution of the variance from the first three principal components surpassed 75% (Figure 4c). Finally, we performed GO analysis (Figure 4i) and KEGG analysis (Figure 4j) for 1310 key genes. In our analysis of up-regulated enrichment, we observed a significant involvement of these key genes in the anti-tumor immune response, such as: the activation, regulation, and proliferation of B cells, T cells, and macrophages; induction of tumor immunity by MHC-I/II molecules and inhibition of tumor cell proliferation by such molecules; PD-1/PD-L1 can be employed to elicit immune cell activation within tumor sites, thereby fostering anti-tumor immune responses. During down-regulation enrichment analysis, these key genes were mainly linked to the weakening of the anti-tumor immune pathway, leading to tumor proliferation and migration, such as: the weakening of cell adhesion function; the inactivation of anticancer substances by P450; the inhibition of the infiltration of immune cells into tumors by GABA to promote tumor growth in vivo. Therefore, the 1310 key genes screened by WGCNA modularization of immunoactivity grouping have a certain reference value for the immune response against ICC, as well as the immune escape, proliferation, and migration of ICC.

### 3.4. Different Subpopulations of Infiltrating Immune Cells in ICC

**Figure 5:**
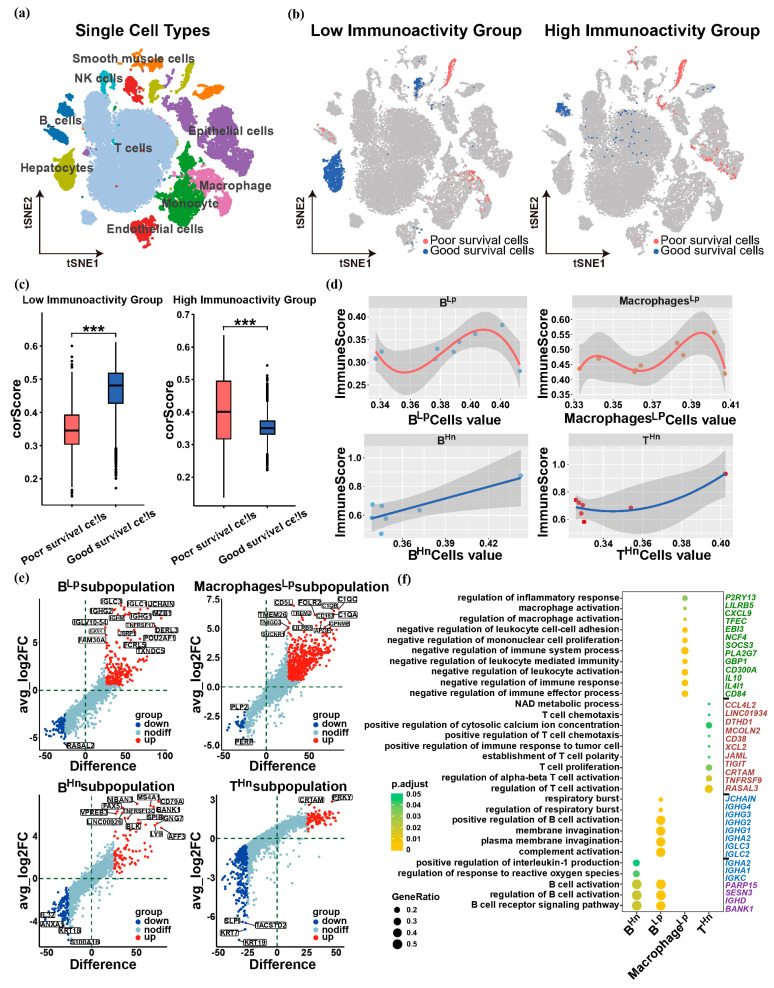
Infiltrating immune cells in intrahepatic cholangiocarcinoma can identify four subpopulations. (**a**) t-SNE plot of cells from ICC, which identified 11 major cell clusters expressing known marker genes. (**b**) In the low immunoactivity group, some B cells and some macrophages have a poor survival rate; these B cells are named as $B^{Lp}$, and the macrophages are named as ${Macrophages}^{Lp}$. In the high immunoactivity group, some B cells and some T cells had better survival rate, which are designated $B^{Hn}$ and $T^{Hn}$. Cells in the gray area were not significantly correlated with the survival rate of specific phenotypes, with *p* value > 0.05. (**c**) Differences between cells with poorer survival and better survival in the low-high immunoactivity groups. Where *p* value ≤ 0.001, ***. (**d**) The immune scores of $B^{Lp}$ and ${Macrophages}^{Lp}$ were generally low, and showed a downward trend as their single-cell expression value increased. The overall immune scores of $B^{Hn}$ and $T^{Hn}$ are higher, and show an upward trend as their single-cell expression value increased. (**e**) Differential genes of four immune cell subpopulations. (**f**) Characteristics of four immune cell subpopulations and their corresponding key genes.

In order to examine the cellular heterogeneity and genetic markers in ICC, 36,558 single cells were selected and retained from a total 55,359 cells for further analysis after quality control and normalization. We then performed dimensionality reduction and clustering using PCA and t-SNE. We identified 11 major cell clusters expressing known marker genes, which include neurons, endothelial cells, hepatocytes, NK cells, T cells, B cells, smooth muscle cells, epithelial cells, neutrophils, monocytes, and macrophages (Figure 5a). The Scissor tool was employed to merge the data from the low immunoactivity group and the high immunoactivity group with the single-cell data for subsequent analysis, respectively. It can be observed that in the low immunoactivity group, some B cells and some macrophages have a poor survival rate. We named these B cells as $B^{Lp}$, and the macrophages as ${Macrophages}^{Lp}$. In the high immunoactivity group, some B cells and some T cells had better survival rate, which we designated $B^{Hn}$ and $T^{Hn}$, respectively (Figure 5b). Here, we believe that from the perspective of immunity, four subpopulations of infiltrating immune cells in cholangiocarcinoma can be identified: $B^{Lp}$, ${Macrophages}^{Lp}$, $B^{Hn}$, and $T^{Hn}$. To verify the accuracy of the joint analysis, we analyzed the difference between single-cell signatures of better or poorer survival in the low immunoactivity group, as well as analyzing the difference between single-cell signatures of better or poorer survival in the high immunoactivity group (Figure 5c). At the same time, we observed the correlation analysis between the immune score of the transcriptome data and $B^{Lp}$, ${Macrophages}^{Lp}$, $B^{Hn}$, $T^{Hn}$ in the joint analysis, respectively. It could be seen that the immune scores of $B^{Lp}$ and ${Macrophages}^{Lp}$ were generally low, and showed a downward trend as their single-cell expression value increased. $B^{Hn}$ and $T^{Hn}$ exhibited higher immune scores, which displayed an ascending pattern in conjunction with the increase in their respective single-cell expression values (Figure 5d). This is consistent with the poor survival rate of $B^{Lp}$ and ${Macrophages}^{Lp}$ mentioned above, and the better survival rate of $B^{Hn}$ and $T^{Hn}$. We performed differential analysis of $B^{Lp}$, ${Macrophages}^{Lp}$, $B^{Hn}$, and $T^{Hn}$ to screen out significant genes (Figure 5e), and analyzed and observed the enrichment pathways of significant genes in the four subpopulations. We tried to find characteristic properties that are unique to each subpopulation (Figure 5f).

In the $B^{Lp}$ subpopulation, *IGLC1-3*, *IGHG1-4*, *IGHA1-2*, etc. were highly expressed, and the characteristics of plasma membrane invagination, complement activation, and respiratory burst were related to and representative of the $B^{Lp}$ subpopulation. In the case of the above pathways, we believe that the cell membrane of the $B^{Lp}$ subpopulation loses its homeostasis and enhances its fluidity. Meanwhile, during an immune response, respiratory burst (this phenomenon is primarily observed in myeloid cells, particularly macrophages and neutrophils) releases reactive oxygen species (ROS), which can create an oxidative environment. This oxidative environment can impact the activation and function of B cells, influencing their ability to produce antibodies and participate in immune response [58]. The duality of complement may inhibit anti-tumor immunity in the $B^{Lp}$ subpopulation. These phenomena significantly contribute to the occurrence, progression, and migration of tumors. Consequently, the $B^{Lp}$ subpopulation exhibits a propensity to facilitate the proliferation, infiltration, and metastasis of ICC.

In the $B^{Hn}$ subpopulation, *BANK1*, *SESN3*, *IGHD*, *PARP15*, etc. were highly expressed, regulation of response to reactive oxygen species, positive regulation of interleukin-1 (IL-1) production, and the B cell receptor signaling pathway were related to and representative of the $B^{Hn}$ subpopulation. Despite previous research demonstrating the inhibitory effects of IL-1 on B cell proliferation and the production of immunoglobulin-secreting cells, IL-1 can negatively regulate B cell activation [59]. However, with the emergence of the B cell receptor signaling pathway, we speculate that it is likely that mature B cells enter an antigen-dependent developmental stage, and they differentiate into activated B cells, memory B cells, and plasma cells after receiving antigen stimulation, thereby generating an anti-tumor immune response effect. Moreover, for the regulation of ROS, it is also a good illustration that $B^{Hn}$ subpopulation avoids the influence of ROS on it. This is also the reason why the aforementioned $B^{Hn}$ subpopulation has a relatively good survival rate. Therefore, the $B^{Hn}$ subpopulation is prone to produce anti-tumor immune response properties.

In the ${Macrophages}^{Lp}$ subpopulation, *CD84*, *IL4I1*, *IL10*, etc. were highly expressed. Macrophage activation occurs in the ${Macrophages}^{Lp}$ subpopulation, which can be antitumor. However, many negatively regulated pathways have emerged, such as negative regulation of immune effector process, negative regulation of leukocyte-mediated immunity, and negative regulation of mononuclear cell proliferation, and there is also an inflammatory response, which can promote tumor invasion and metastasis. So, the ${Macrophages}^{Lp}$ subpopulation appears to be less effective for ICC in resisting tumor invasion and metastasis.

In the $T^{Hn}$ subpopulation, *CD38*, *NCF4*, *CRTAM*, etc. are highly expressed, T cell activation, T cell polarity, T cell chemotaxis, positive regulation of cytosolic calcium ion concentration, and NAD metabolic process related to the $T^{Hn}$ subpopulation are correlated and representative. Chemotaxis plays a crucial role in directing T cell migration and recruitment to specific sites [60]. This helps T cells efficiently destroy tumor cells. In addition, calcium signaling is critical for T cell activation, cytokine production, and immune responses [61]. NAD can also influence T cell polarization and potentially promote specific T cell phenotypes [62]. So, the $T^{Hn}$ subpopulation tends to kill tumor cells for ICC.

The significant genes unique to each immune cell subpopulation were obtained by intersecting the genes of each subpopulation with the 1310 module genes mentioned previously (Table 1).

## 4. Discussion

**Figure 6:**
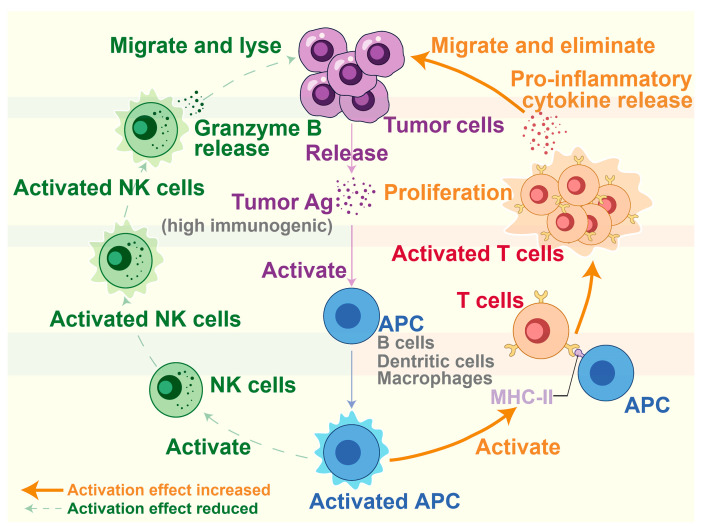
Model of ICC’s immune sensing and response. After ICC tumor cells release high immunogenic antigens to activate APCs, two immune response pathways will appear: (**right**) the effect of activating T cells is enhanced; (**left**) the effect of activating NK cells is weakened.

This study aimed to examine the activity of infiltrating immune cells in ICC, revealing that B cells, macrophages, and T cells exhibited significantly heightened levels of activity in comparison to other immune cell types. In addition, we identified four subpopulations of ICC-infiltrating immune cells as well as several immune-related prognostic labels. Ultimately, we present a novel immune sensing and response model for ICC, drawing upon our existing understanding of its immune sensing and response (Figure 6).

We have found that B cells, macrophages, and T cells in the tumor site of ICC were significantly different from those in the paracancer site, and their proportion of cell abundance was high, while NK cells were not obvious. During the elimination phase of immune surveillance, ICC tumor cells release highly immunogenic antigens that activate APCs [63,64]. Activated APCs serve two functions: (a) to activate NK cells so that they migrate to tumor sites and release molecules such as granzymes, which dissolve and eradicate tumors, while at the same time, NK cells also promote the activation of APCs [65,66,67,68,69]; (b) to cause activated T cells to migrate to the tumor site to release proinflammatory cytokines and multiply by presenting tumor antigens on the MHC-II, which causes death and elimination of tumor cells [70,71,72,73,74]. In our study of infiltrating immune cells in ICC, we found that APCs activated during the immune sensing and response may be less effective in activating NK cells and more effective in activating T cells (Figure 6).

The immunoactivity of infiltrating immune cells in ICC may be related to and affected by the four gene modules (1310 in total) screened out. We found that these genes are related to the anti-tumor immune response, including the activation, regulation, and proliferation of B cells, T cells, and macrophages. The induction of tumor immunity is facilitated by the presence of MHC-I/II molecules, which also exert a certain degree of inhibition on tumor proliferation. It is interesting to note that these genes appear to be associated with immune escape and proliferation migration of ICC. In the pathway, we have seen the appearance of PD-1/PD-L1, P450, and GABA, which can inhibit the immune response, weaken the infiltration of immune cells into tumor cells, inactivate anticancer substances, and promote tumor cell proliferation, and so on. At the same time, the attenuation of cell adhesion function leads to the migration of tumor cells.

Subsequently, we identified four subpopulations: $B^{Lp}$, $B^{Hn}$, ${Macrophages}^{Lp}$, and $T^{Hn}$. *IGLC1-3* [75], *IGHG1-4* [76,77,78], *IGHA1-2* [77], etc. are highly expressed in $B^{Lp}$. The respiratory burst impacts activation and function of $B^{Lp}$, and the activation of complement inhibits the immune response, thereby promoting tumor growth and migration; and *BANK1* [79], *SESN3*, *IGHD* [80], *PARP15*, etc. in the $B^{Hn}$ are highly expressed, and there is a phenomenon that IL-1 negatively regulates B cell activation. But the $B^{Hn}$ subpopulation belongs to mature B cells; after being stimulated by an antigen, they will carry out an antitumor immune response. *CD84* [81], *IL4I1* [82], *IL10* [83], etc. are highly expressed in ${Macrophages}^{Lp}$. These cells are associated with the inflammatory response and are implicated in multiple negatively regulated pathways that can facilitate the invasion and metastasis of tumor cells; in $T^{Hn}$, *CD38* [84], *NCF4*, *CRTAM* [85], etc. are highly expressed. Chemotaxis, polarity, and calcium signaling lead $T^{Hn}$ to have the function of killing tumor cells. The infiltrating immune cells described above are heterogeneous.

Of course, our research content may seem to be contrary to some existing theoretical studies (such as T cell exhaustion [86]), and there may be some opposing views. This article mainly focuses on the immune sensing and response stages in the TME of ICC. This is different from the fact that T cell exhaustion mainly occurs in the immune effector stage [87,88,89]. Our study reflects the early stage of the immune mechanism, that is, the effect of APCs on NK cells and T cells. At this stage, T cells usually have not experienced long-term antigen stimulation, so their functional state may not be completely consistent with the T cell exhaustion phenomenon in chronic infection or late tumor progression [87,90]. Our results show that APCs become more effective in activating T cells and weaker in activating NK cells. This is not contradictory to the T cell exhaustion phenomenon observed in the immune effector stage. On the contrary, they jointly reflect the dynamics and stage specificity of the immune mechanism and reveal the complex behavior of immune cells. Our research may provide a new perspective for understanding the dynamic changes of immune cells in ICC.

Some limitations of this study cannot be ignored. Although we analyzed the transcriptomic sample data of GDC and the single-cell data of NCBI, in the absence of more comprehensive data, such as spatial transcriptomics [91] and image data [92], it is impossible to describe the immune pattern within the TME of ICC more comprehensively, and we did not analyze its clinicopathological features. Furthermore, the limited quantity of cells belonging to the four immune cell subpopulations $B^{Lp}$, ${Macrophages}^{Lp}$, $B^{Hn}$, $T^{Hn}$ identified in the scRNA-seq data hindered our ability to evaluate their molecular characterization at the individual cell level comprehensively and precisely. This issue could potentially be resolved in future investigations employing larger sample sizes that encompass these four ICC-infiltrating immune cell subpopulations. Additionally, the absence of functional data [93,94,95] in our study constrains our comprehension of the molecular mechanisms governing ICC-infiltrating immune cell subpopulations. In the future, further animal studies can potentially provide clarification on this issue and validate our findings.

## 5. Conclusions

In summary, our findings indicate that the activation of T cells by APCs in the ICC immune sensing and response is enhanced, while the activation of NK cells is weakened. Meanwhile, we identified four immune cell subpopulations in low-high immunoreactivity groups, namely $B^{Lp}$, ${Macrophages}^{Lp}$, $B^{Hn}$, and $T^{Hn}$, as well as key genes associated with each subpopulation. It is important to understand the immune status within the TME of ICC in order to guide immunotherapy and prognosis in the future.

## Figures and Tables

**Table 1:** Intersection table of modular genes and differential genes of immune cell subpopulations.

Immune Cell Subpopulation	Significant Genes
$B^{Lp}$	*TNFRSF17*, *PIM2*, *ZBP1*, *CPNE5*, *JCHAIN*, *SLC2A5*, *FCRL5*, *MZB1*, *CHST2*, *IGKC*, *IGKV4-1*, *IGLV10-54*, *IGLV3-1*, *IGLC2*, *IGLC3*, *IGHA2*, *IGHG4*, *IGHG2*, *IGHA1*, *IGHG1*, *IGHG3*, *IGHM*, *LINC02384*, *IGHGP*, *AC007569.1*, *AC239799.2*
$B^{Hn}$	*SESN3*, *BANK1*, *PARP15*, *IGHD*
${Macrophages}^{Lp}$	*CD84*, *NCF4*, *IL4I1*, *EBI3*, *LILRB5*, *TFEC*, *GBP1*, *IL10*, *CXCL9*, *SELENBP1*, *PLA2G7*, *CD300A*, *ZNF804A*, *C1QB*, *P2RY13*, *SOCS3*, *FCGR1B*, *AC110995.1*, *LINC01010*, *MRC1*
$T^{Hn}$	*CD38*, *TNFRSF9*, *RASAL3*, *CRTAM*, *XCL2*, *MCOLN2*, *JAML*, *TIGIT*, *DTHD1*, *LINC01934*, *CCL4L2*

## Data Availability

Publicly available datasets were analyzed in this study. The ICC transcriptome samples and their corresponding clinical phenotypes were obtained from the GDC data portal (https://portal.gdc.cancer.gov/ accessed on 1 December 2023) and UCSC Xena portal (https://xena.ucsc.edu/ accessed on 3 December 2023). Single-cell samples were obtained from the NCBI Gene Expression Omnibus (GEO) database (https://www.ncbi.nlm.nih.gov/ accessed on 1 May 2024; GSE138709, GSE159929). The LM22 gene set matrix was obtained from the CIBERSORT website (https://cibersortx.stanford.edu/ accessed on 10 December 2023). The collection of 16 immune-related gene sets contains 929 genes from TISCH2 (http://tisch.comp-genomics.org/ accessed on 10 December 2023). The source code for this study can be obtained from https://gitHub.com/Gadbee0518/Immune-ICC accessed on 26 August 2024.

## Supplementary Materials

The following supporting information can be downloaded at: https://www.mdpi.com/article/10.3390/biology13100816/s1, Comparison of immunoactivity. Principal component analysis [96,97,98].
