# Supplementary material for: Multi-Omics Profiling Unveils the Complexity and Dynamics of Immune Infiltrates in Intrahepatic Cholangiocarcinoma
Source: Biology (Basel). 2024 Oct 11;13(10):816. doi: 10.3390/biology13100816 (PMC11504529; doi:10.3390/biology13100816)
Supplement: Supplementary file 1 [file biology-13-00816-s001.zip › biology-3211760-supplementary.pdf]

Supplementary Materials

Some methods designed by ourselves and others in the main text are introduced and reasoned here. Method 1 is a method designed by us, which aims to evaluate and judge the changes in the immunoactivity of infiltrating immune cells under different conditions; Method 2 is a method designed by others, which provides the basis for gene screening, clustering, single cell analysis and other operations in the main text.

1. Comparison of immunoactivity

We designed a method to estimate and compare immunoactivity. After estimating the immune activity expression value of a single immune cell in each sample through the ssGSEA analysis method, a linear regression analysis was performed on the proportion of the immune cell and its immunoactivity expression value. If there are two regression lines, the angle between them can represent the magnitude of the change from one state to another. The formula is as follows:

$$Y = kX + b, \tag{S1}$$

In formula (1), Y represents the proportion of immune cells, and X represents the expression value of immunoactivity.

$$L(\hat{y}_i, y_i) = (\hat{y}_i - y_i)^2, \tag{S2}$$

Formula (2) represents the error between the predicted value $\hat{y}_i$ and the true value y_i of a single sample i in regression.

$$L(\hat{Y}, Y) = \frac{1}{N}\sum_{i=1}^{N}(\hat{y}_i - y_i)^2 = \frac{1}{N}\sum_{i=1}^{N}[(kx_i + b) - y_i]^2, \tag{S3}$$

In formula (3), N represents the number of samples, that is, there are N data pairs (x_1, y_1), (x_2, y_2), ..., (x_N, y_N).

$$k', b' = \underset{k,b}{argmin}\frac{1}{N}\sum_{i=1}^{N}[(kx_i + b) - y_i]^2, \tag{S4}$$

Formula (4) means finding the parameters k' and b' that minimize the function value of formula (3).

$$\Delta k = k'_T - k'_C, \begin{cases} > 0, & Increased \\ = 0, & Unchanged \\ < 0, & Reduced \end{cases} \tag{S5}$$

The slope k'_C of the regression line in the Control Group may be inter-preted as indicative of immune cell activity in a healthy state, whereas the slope k'_T of the regression line in the Tumor Group may be interpreted as indicative of immune cell activity in a cancerous state. Δk represents the change in immunoactivity after the state transition. $\Delta k > 0$ indicates that the immunoactivity in the healthy state increases to that in the cancerous state; $\Delta k = 0$ means that the immunoactivity does not change from the healthy state to the cancerous state; $\Delta k < 0$ indicates that the immunoactivity in the healthy state decreases to the immune activity in the cancerous state.

2. Principal component analysis

Principal component analysis (PCA) is a statistical method that can reduce the dimensionality of a dataset through dimensionality reduction techniques while retaining as much variability as possible in the original data. PCA is often used for exploratory data analysis and building predictive models [1, 2]. The main steps of PCA are as follows [1, 3]:

Assuming the number of samples is S, and the gene of the sample is used as the feature dimension G, a two-dimensional matrix $[x_1, x_2, \ldots, x_S][f_1, f_2, \ldots, f_G]^T$ will be formed. The mean vector of the sample is calculated as shown in formula (6).

$$\bar{X} = \frac{1}{S}\sum_{i=1}^{S} x_i, \tag{S6}$$

Formula (7) is the centralized processing.

$$X' = X - \bar{X}, \tag{S7}$$

Formula (8) constructs the covariance matrix.

$$H = \frac{1}{S}X'^{T}X', \tag{S8}$$

Perform eigenvalue decomposition on the covariance matrix H to obtain eigenvalues λ_1, λ_2, ..., λ_G, and corresponding eigenvectors v_1, v_2, ..., v_G. Setting the parameter to k means selecting the first k eigenvectors as principal components to form the projection matrix P, and we have formula (9).

$$X'_{reduced} = X'P, \tag{S9}$$

where $X'_{reduced}$ is the dataset after dimensionality reduction.

References

1. Ma, S.; Dai, Y., Principal component analysis based methods in bioinformatics studies. *Brief Bioinform* **2011,** 12, (6), 714-22. https://doi.org/10.1093/bib/bbq090.
2. Elhaik, E., Principal Component Analyses (PCA)-based findings in population genetic studies are highly biased and must be reevaluated. *Sci Rep* **2022,** 12, (1), 14683. https://doi.org/10.1038/s41598-022-14395-4.
3. Yao, F.; Coquery, J.; Le Cao, K. A., Independent Principal Component Analysis for biologically meaningful dimension reduction of large biological data sets. *BMC Bioinformatics* **2012,** 13, 24. https://doi.org/10.1186/1471-2105-13-24.
